# 4016 Combined Eating Disorder and Weight-Loss Online Guided-Self Help Intervention: Updated Results from an Ongoing Pilot Study

**DOI:** 10.1017/cts.2020.192

**Published:** 2020-07-29

**Authors:** Grace Elise Monterubio, Ellen E. Fitzsimmons-Craft, Denise E. Wilfley

**Affiliations:** 1 Washington University in St. Louis, Institute Of Clinical and Translational Sciences

## Abstract

OBJECTIVES/GOALS: Aims 1&2: Develop (1) and implement (2) online, guided self-help intervention for ED psychopathology and weight reduction. Aim 3: Follow-up to track remission of ED psychopathology and symptoms and WL maintenance at end of treatment and 6-months. METHODS/STUDY POPULATION: N = 60 college students meeting criteria (clinical/sub-clinical binge-type ED with BMI > 25) will complete a baseline survey and then will be randomized into a condition. Students in the intervention group (n = 30) will be offered 8 weeks of an online, guided self-help intervention for ED and WL. Students in the control group (n = 30) will receive an email message to seek support from Student Health Services. All participants will receive follow-ups 9 weeks and 6 months after baseline. Data analysis will compare Eating Disorder Examination Questionnaire (EDE-Q) scores and WL (change in BMI) at all three time-points. Group comparisons will be assessed via two-way mixed-model ANOVA. RESULTS/ANTICIPATED RESULTS: Recruitment is still ongoing. Data collected by the time of the conference will be presented on the poster. DISCUSSION/SIGNIFICANCE OF IMPACT: Online, guided self-help interventions have been used for WL, as well as for treatment of EDs separately, but no program exists to manage these commonly comorbid conditions concurrently. Thus, this pilot study will examine the effectiveness of combined programs to breach this treatment gap.

